# Clinical characteristics of elephant endotheliotropic herpesvirus (EEHV) cases in Asian elephants (*Elephas maximus*) in Thailand during 2006–2019

**DOI:** 10.1080/01652176.2021.1980633

**Published:** 2021-09-25

**Authors:** Yaoprapa Yun, Supaphen Sripiboon, Kidsadagon Pringproa, Phongsakorn Chuammitri, Veerasak Punyapornwithaya, Khajohnpat Boonprasert, Pallop Tankaew, Taweepoke Angkawanish, Kittikul Namwongprom, Orapun Arjkumpa, Janine L. Brown, Chatchote Thitaram

**Affiliations:** a Faculty of Veterinary Medicine, Center of Elephant and Wildlife Research, Chiang Mai University (FVM-CMU), Chiang Mai, Thailand; b Graduate Program in Veterinary Science, Faculty of Veterinary Medicine, Chiang Mai University, Chiang Mai, Thailand; c Faculty of Veterinary Medicine, Department of Large Animal and Wildlife Clinical Sciences, Kasetsart University (FVM-KU), Kamphaeng Saen Campus, Nakornpathom, Thailand; d Faculty of Veterinary Medicine, Department of Veterinary Biosciences and Public Health, Chiang Mai University, Chiang Mai, Thailand; e Faculty of Veterinary Medicine, Veterinary Public Health and Food Safety Centre for Asia Pacific, Chiang Mai University, Chiang Mai, Thailand; f Elephant Hospital, National Elephant Institute (NEI), Forest Industry Organization, Lampang, Thailand; g Phra Nakhon Si Ayutthaya Provincial Livestock Office, Phra Nakhon Si Ayutthaya Province, Thailand; h Center for Species Survival, Smithsonian Conservation Biology Institute (SCBI), Front Royal, VA, USA; i Department of Companion Animal and Wildlife Clinics, Faculty of Veterinary Medicine, Chiang Mai University, Chiang Mai, Thailand

**Keywords:** Asian elephant, *Elephas maximus*, elephant endotheliotropic herpesvirus (EEHV), clinical characteristics, retrospective study, Thailand

## Abstract

**Background:**

Elephant endotheliotropic herpesvirus causes a hemorrhagic disease (EEHV-HD) that is a major cause of death in juvenile Asian elephants with EEHV1 and EEHV4 being the most prevalent.

**Aim:**

To perform a retrospective clinical data analysis.

**Methods:**

Records of a total of 103 cases in Thailand confirmed by polymerase chain reaction (PCR) on blood and/or tissue samples.

**Results:**

The severity of clinical signs varied among EEHV subtypes. EEHV1A was the most prevalent with 58%, followed by EEHV4 with 34%, EEHV1B with 5.8% and EEHV1&4 co-infection with 1.9%. Overall case fatality rate was 66%. When compared among subtypes, 100% case fatality rate was associated with EEHV1&4 co-infection, 83% with EEHV1B, 75% with EEHV1A, and the lowest at 40% for EEHV4. Calves 2- to 4-year old were in the highest age risk group and exhibited more severe clinical signs with the highest mortality. Majority of cases were found in weaned or trained claves and higher number of cases were observed in rainy season. A gender predilection could not be demonstrated. Severely affected elephants presented with thrombocytopenia, depletion of monocytes, lymphocytes and heterophils, a monocyte:heterophil (M:H) ratio lower than 2.37, hypoproteinemia (both albumin and globulin), severe grade of heterophil toxicity, and low red blood cell counts and pack cell volumes. Survival was not affected by antiviral drug treatment in the severely compromised animals.

**Conclusion:**

Early detection by laboratory testing and aggressive application of therapies comprising of supportive and anti-viral treatment can improve survival outcomes of this disease.

## 1. Introduction

Elephant endotheliotropic herpesvirus is a highly fatal hemorrhagic disease (EEHV-HD), discovered using PCR in 1999 (Richman et al. 1999) and today is the leading cause of death in juvenile Asian elephants (*Elephas maximus*) aged 1–8 years (Stanton et al. 2012; Richman et al. 2014; Wilkie et al. 2014). Initial clinical signs are not always apparent, but can include lethargy, anorexia, and fever. As the disease progresses, edema of the head and neck, and cyanosis of the tongue are present in many cases, which ultimately results in a fatal outcome within days of clinical signs with an 80–85% case fatality rate (Sripiboon et al. 2013; Richman et al. 2014; Long et al. 2016). EEHV1 and EEHV4 are the two most common types associated with mortality in Asian elephants, with only a few cases of successful treatment using antiviral drug and supportive treatments (Sripiboon et al. 2013, 2017; Zachariah et al. 2013; Richman et al. 2014; Dastjerdi et al. 2016; Long et al. 2016; Fuery, Browning, et al. 2016; Khammesri et al. 2021).

The estimated population of captive elephants in Thailand in 2019 was 3845 with 405 being less than 8 years of age [Data from the National Elephant Institute (NEI)], the high-risk age group for EEHV. Between 2006 and 2018, of 58 EEHV-HD cases in Thailand, 76% were EEHV1, 20% were EEHV4, and 4% were EEHV1&4 co-infection. The overall mortality rate was 69% (Boonprasert et al. 2019). However, although molecular diagnosis by PCR and serology tests have been available for EEHV detection for more than 18 years, information on clinical characteristics, epidemiology, pathogenesis, routes of viral transmission, and the role of factors in disease risk are limited (Fickel et al. 2001; Garner et al. 2009; Fuery et al. 2020). Furthermore, lack of proper data collection and records, inadequate elephant health monitoring systems, and limited access to PCR equipment for real-time diagnosis in range countries have contributed to the unchecked spread of this disease, including in Thailand (Fickel et al. 2001; Bennett et al. 2015; Barman et al. 2017; Boonprasert et al. 2019).

Thailand’s first EEHV case was reported in 2006 (Sripiboon et al. 2013; Boonprasert et al. 2019) and by the end of 2019, 103 were PCR-confirmed, representing the highest number of EEHV cases in the world. In this study, a retrospective data analysis of these cases was conducted to provide a better understanding of the epidemiology, pathogenesis, and clinical characteristics of this lethal disease.

## 2. Materials and methods

### 2.1. Study design

Clinical data were obtained from 103 PCR-confirmed EEHV cases using blood and/or tissue samples throughout Thailand from January 2006 to December 2019. Animals were grouped based on the severity of clinical signs: (1) subclinical - no clinical signs present; (2) non-severe - minimal clinical signs, including lethargic with or without appetite lost, fever, or diarrhea; and (3) severe - clinical signs of EEHV-HD such as facial or neck edema, tongue cyanosis, bloody diarrhea, or death. Case fatality rate was calculated as the total number of elephants that died from EEHV divided by the total number of elephants with a positive PCR result and with clinical signs present.

Demographic information was collected on EEHV PCR-positive cases and included elephant name or microchip number, gender, age, season, location, and region, EEHV PCR test method and results, blood profiles, clinical signs (lethargic, fever, diarrhea, bloody diarrhea, facial edema, and tongue cyanosis), existence of medical records, general husbandry such as weaning, and training status in the month prior to a positive EEHV result.

### 2.2. Diagnostic methods

All EEHV positive cases were confirmed by conventional and/or real-time quantitative PCR methods from blood and/or tissue samples of infected elephants. Tests were conducted at one of three EEHV diagnostic laboratories in Thailand: Faculty of Veterinary Medicine, Chiang Mai University (FVM-CMU); Faculty of Veterinary Medicine, Kasetsart University (FVM-KU); or the NEI. Conventional PCR protocols were followed as described by Latimer et al. (2011), while quantitative real-time PCR protocols were based on Stanton et al. (2010). EEHV1 positive cases were sequenced to discriminate EEHV1 subtypes as described by Stanton et al. (2012).

Hematology tests were conducted at FVM-CMU on a BC-5300 vet auto hematology analyzer (Mindray, Shenzhen, P. R. China) within 24 h after blood collection, while white blood cell differential counts were done manually on Wright-Giemsa-stained blood smears.

### 2.3. Statistical analysis

Count and percentage data were expressed as both continuous and categorical variables. No imputation for not available (NA) data was made. Because the retrospective data did not derive the population of EEHV cases from random sampling, all statistics were descriptive only. We used QGIS, version 3.14.1 (QGIS 2020) to plot the numbers of cases on the Thailand map with location. All analyses were performed using R statistical software version 1.1.414 (R-studio 2019).

## 3. Results

### 3.1. Demographic and clinical characteristics

**Figure 1.**
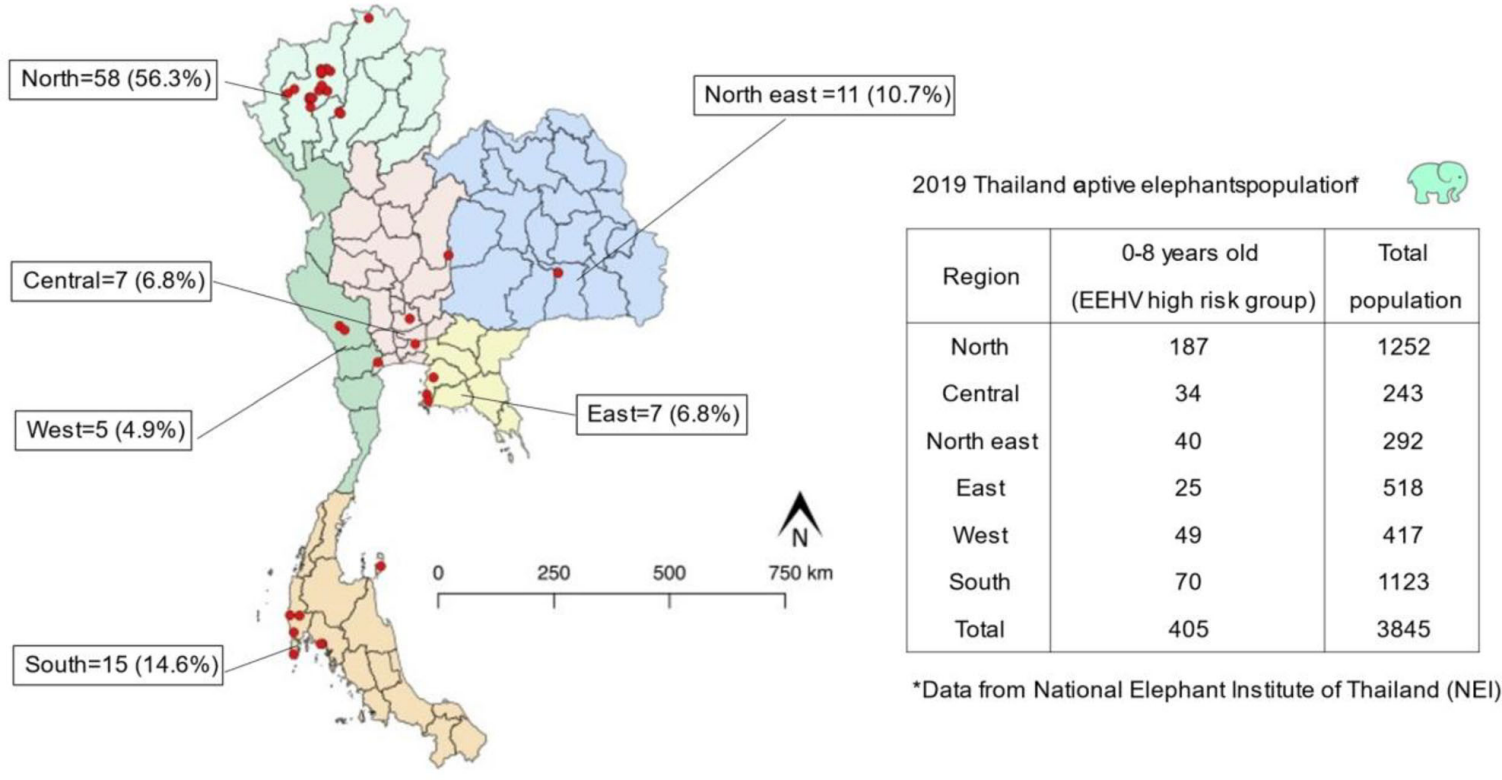
Distribution of 103 PCR-confirmed EEHV cases in Thailand during 2006–2019. Location of individual elephants is indicated by red dots. The text box indicates the number of elephants in the high risk age group (0- to 8-year old) out of the total number of captive elephants in Thailand in 2019.

From the 103 PCR-confirmed cases, the greatest numbers were reported in northern Thailand 58/103 (56%), with the rest distributed among the southern (14/103, 15%), northeastern (11/103, 11%), central (7/103, 6.8%), eastern (7/103, 6.8%), and western (5/103, 4.9%) regions (Figure 1). There was no relationship between region and disease severity (Table 1).

**Table 1.** Demographic and clinical characteristics of PCR-confirmed EEHV cases in Thailand during 2006–2019 by severity of clinical signs.

Demographic and characteristics	Number of cases	Severity of clinical sign		
		Subclinical	Non-severe	Severe
Died	46/89(51.6)	0	0	46/46(100)
Survived	43/89(48.3)	12/43(27.9)	25/43(58.1)	6/43(13.9)
NA	14			
Sex				
Female	54/99(54.5)	8/54(1.5)	13/54(24.1)	26(48.1)
Male	45/99(45.5)	5/45(11.1)	15/45(33.3)	20/45(44.4)
NA	4			
Age (y)				
0–4	66/87(75.9)	7/66(10.6)	21/66(31.8)	38/66(57.6)
>4–8	12/87(13.8)	2/12(16.7)	3/12((25.0)	7/12(58.3)
>8–12	7/87(8.0)	3/7(42.9)	3/7(42.9)	1/7(14.2)
>12	2/87(2.3)	1/2(50.0)	1/2(50.0)	0
NA	16			
Laboratory findings				
Thrombocytopenia	15/24(62.5)	3/9(33.3)	5/8(62.5)	7/7(100)
Heteropenia	9/24(37.5)	0	2/8(25.0)	7/7(100)
Monocytopenia	12/24(50.0)	0	5/8(62.5)	7/7(100)
Lymphopenia	12/24(50.0)	0	5/8(62.5)	7/7(100)
Monocyte:heterophil ratio < 2.37	12/24(50.0)	0	5/8(62.5)	7/7(100)
Monocytosis	6/24(25.0)	4/9(44.4)	2/8(25.0)	0
Lymphocytosis	9/24(37.5)	7/9(77.8)	2/8(25.0)	0
Heterophilia	8/24(33.3)	3/9(33.3)	5/8(62.5)	0
Heterophil left-shifting	12/24(50.0)	4/9(44.4)	5/8(62.5)	3/7(42.9)
Heterophil toxicity	14/24(58.3)	2/9(22.2)	5/8(62.5)	7/7(100)
Reactive lymphocytes	9/24(37.5)	7/9(77.8)	2/8(25.0)	0
Activated monocytes	8/24(33.3)	3/9(33.3)	4/8(50.0)	1/7(14.3)
Depletion of RBC count and PCV	9/24(37.5)	0	2/8(25.0)	7/7(100)
Hypoproteinemia	9/24(37.5)	0	2/8(25.0)	7/7(100)
NA	79			
Weaning status				
Nursing	20/78(25.6)	5/20(25.0)	5/20(25.0)	10/20(50.0)
Weaned	58/78(74.4)	7/58(12.1)	23/58(39.6)	28/58(48.3)
NA	25			
Training status				
No training	29/75(38.7)	10/29((34.5)	6/29(20.1)	13/29(44.8)
Trained	46/75((61.3)	3/46(6.5)	20/46(43.5)	23/46(50.0)
NA	28			
Region				
North	56/88(63.6)	19/56(33.9)	24/56(42.8)	22/56(39.3)
Central	5/88(5.7)	0	2/5(40.0)	3/5(60.0)
East	4/88(4.5)	0	0	4/4(100)
Northeast	9/88(10.2)	2/9(22.2)	0	7/9(77.8)
West	2/88(2.3)	0	0	2/2(100)
South	12/88(13.6)	1/12(8.3)	2/12(16.7)	9/12(75.0)
NA	15			
Season				
Summer (16th February–15th May)	15/86(17.4)	0	7/15(46.7)	8/15(53.3)
Rainy (15th May–15th October)	41/86((47.7)	4/41(9.7)	15/41(36.6)	22/41(53.7)
Winter (16th October–15th February)	30/86(34.9)	9/30(30.0)	5/30(16.7)	0
NA	17			

Values in parentheses indicate percent by group. NA: not available.

**Figure 2.**
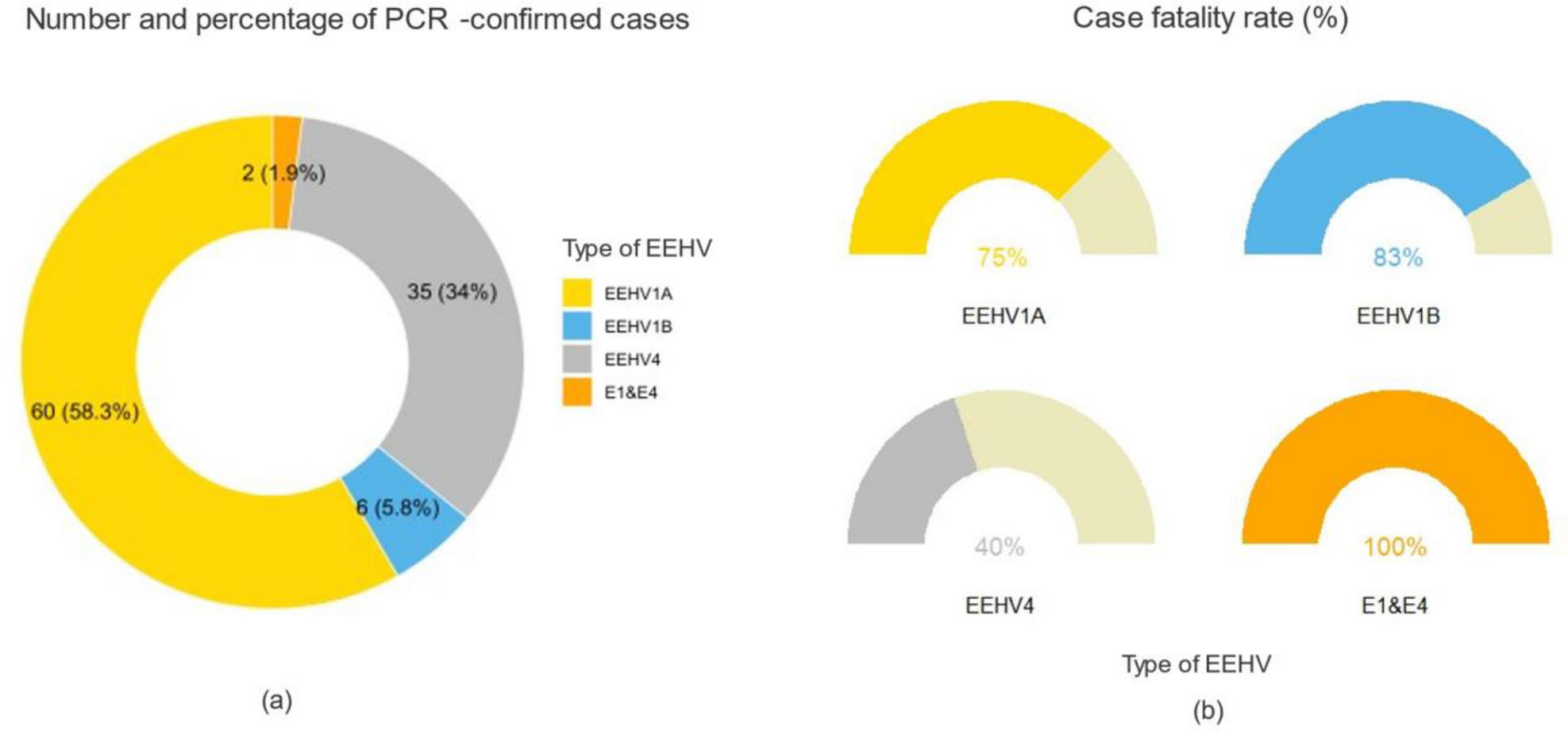
Number and percentage of PCR-confirmed EEHV types in Thailand from 2006 to 2019 out of a total of 103 cases (a), and case fatality rate for 91 cases (b).

EEHV1A was the most prevalent with 60 cases (58%), followed by EEHV4 with 35 cases (34%), EEHV1B at 6 cases (5.8%), and EEHV1&4 co-infection at 2 cases (1.9%) (Figure 2(a)). Case fatality rate was calculated from 91 cases in non-severe and severe clinical groups; overall case fatality rate was 66% (60/91). When compared among subtypes, 100% case fatality rate was associated with EEHV1&4 co-infection, 83% with EEHV1B, 75% with EEHV1A, and the lowest at 40% for EEHV4 (Figure 2(b)).

**Figure 3.**
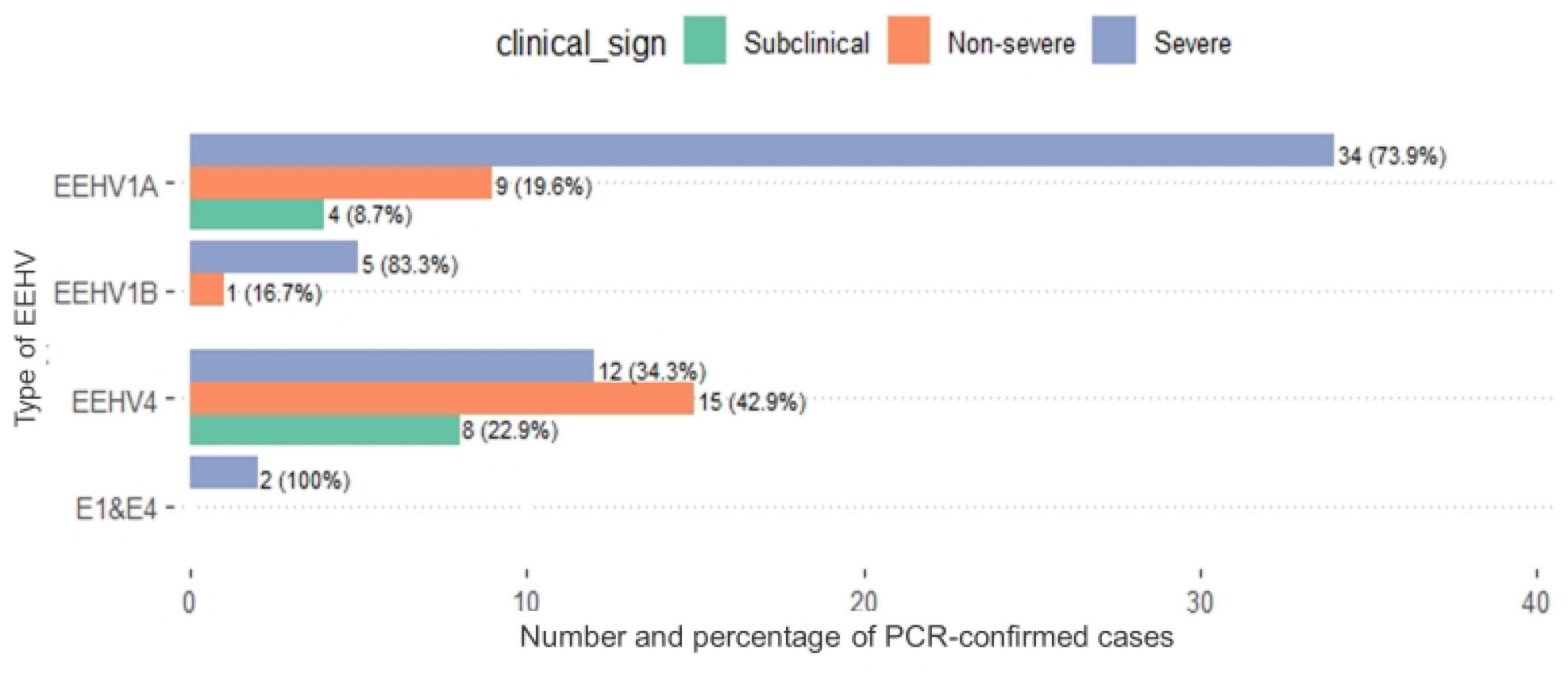
Data for 89 PCR-confirmed EEHV cases in Thailand grouped by type and clinical signs (1) subclinical group = 12/89 (14%), (2) non-severe clinical group = 25/89 (28%), and (3) severe clinical group =52/89 (58%).

**Figure 4.**
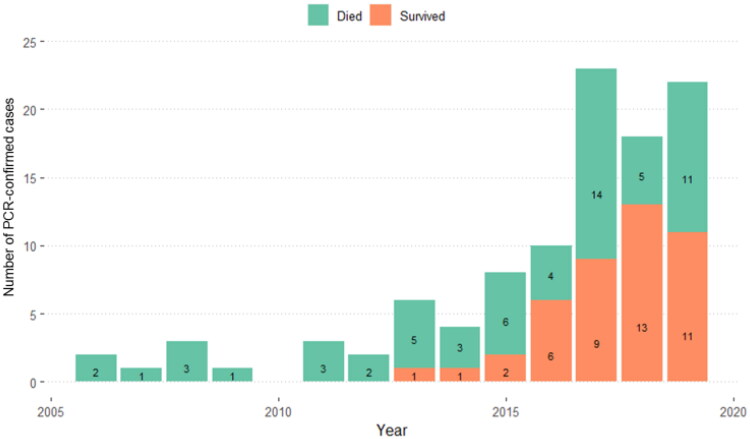
Number of PCR-confirmed EEHV cases in Thailand and how many died or survived by year during 2006–2019, the total number of cases was 103.

Of 89 cases with data (NA = 14), the highest number was found in the severe clinical group at 52/89 (58.4%), second was the non-severe clinical group at 25/89 (28.1%), while the lowest number was in the subclinical group at 12/89 (13.5%). When comparing subtypes with clinical signs, from 52 cases in the severe clinical group, EEHV1A was responsible for the highest number at 34 cases. Among six cases of EEHV1B, five presented with severe clinical signs. Both cases of EEHV1&4 co-infection were in the severe group. In non-severe and subclinical groups, EEHV4 had the highest number at 15 cases in the non-severe and 8 cases in the subclinical groups (Figure 3). From Table 1, all 46 dead elephants were in the severe clinical group, while there was no mortality in the non-severe clinical group. Of 43 surviving cases, there were 12/43 (27.9%) in the subclinical group, 25/43 (58.1%) in the non-severe group, and only 6/43 (13.9%) survived when severe clinical signs presented. Compared to data from 2006 to 2012, the number of cases has increased since 2016, similar to survival rates. The highest number of cases was reported in 2017 with a total number of 23 animals (Figure 4).

**Figure 5.**
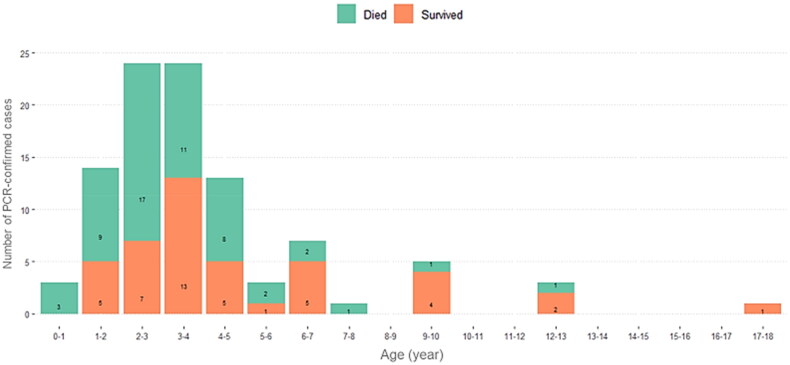
Summary of 98 PCR-confirmed EEHV cases in Thailand that died or survived by age of infection between 2006 and 2019.

When age classes were considered, data from 98 EEHV cases were available (NA = 5). EEHV infections occurred in elephants 0 to 18 years of age. Overall, the majority were observed in calves 1–5 years of age, with the highest number of cases and mortality found among those aged 2- to 3- and 3- to 4-year old. Fewer cases and lower mortality presented in elephants older than 7-year old (Figure 5).

Only 87 out of 103 cases were available to compare between age groups and clinical signs. Most of the cases in elephants 0- to 4- and >4- to 8-year old were in the severe clinical group at 38/66 (58%) and 7/12 (58%), respectively. By contrast, only one case with severe clinical signs was found in the age group >8–12 years. The rest of the cases in the older age groups were divided among subclinical and non-severe clinical groups (Table 1).

Higher numbers of EEHV cases were found in calves that had been weaned (58/78, 74.4%) or trained (46/75, 61.3%) within the previous 30 days. There was no difference between sexes; male elephant cases were 45/99 (46%) while 54/99 (55%) cases occurred in females (Table 1).

### 3.2. Season

**Figure 6.**
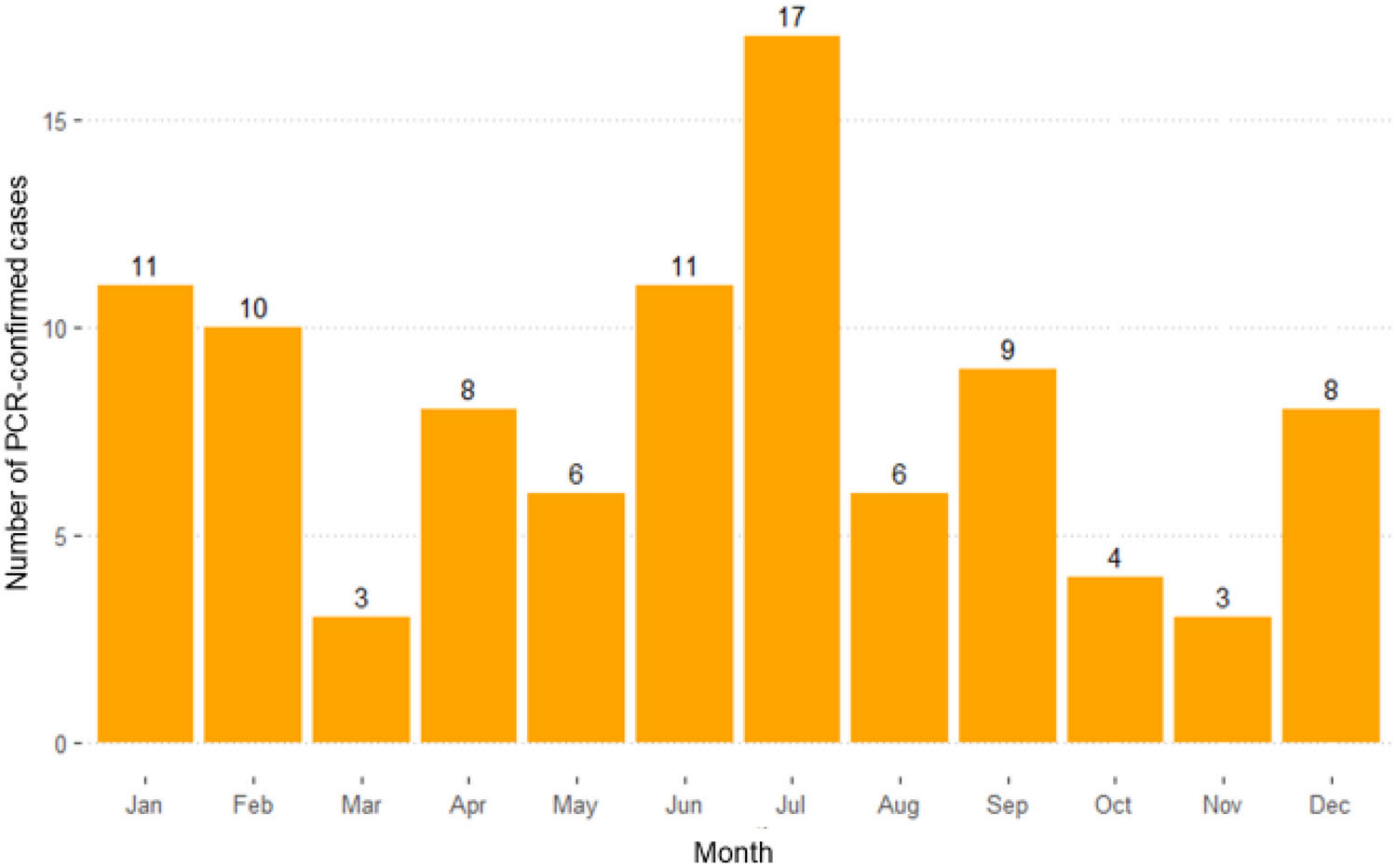
Number of PCR-confirmed EEHV cases by month of infection in Thailand between 2006 and 2019. The total number of cases was 96.

There were 96 cases where data on the month of EEHV infection were available (NA = 7). The three highest case numbers were in July (17/96), June (11/96), and January (11/96) (Figure 6). When data were analyzed by season (*N* = 86; NA = 17), the rainy season (15th May–16th October) consisted of the highest number of cases at 41/86 (48%), followed by winter (16th October–15th February) at 30/86 (35%), and then summer (16th February–15th May) at 15/86 (17%) (Table 1).

### 3.3. Clinical outcomes

**Figure 7.**
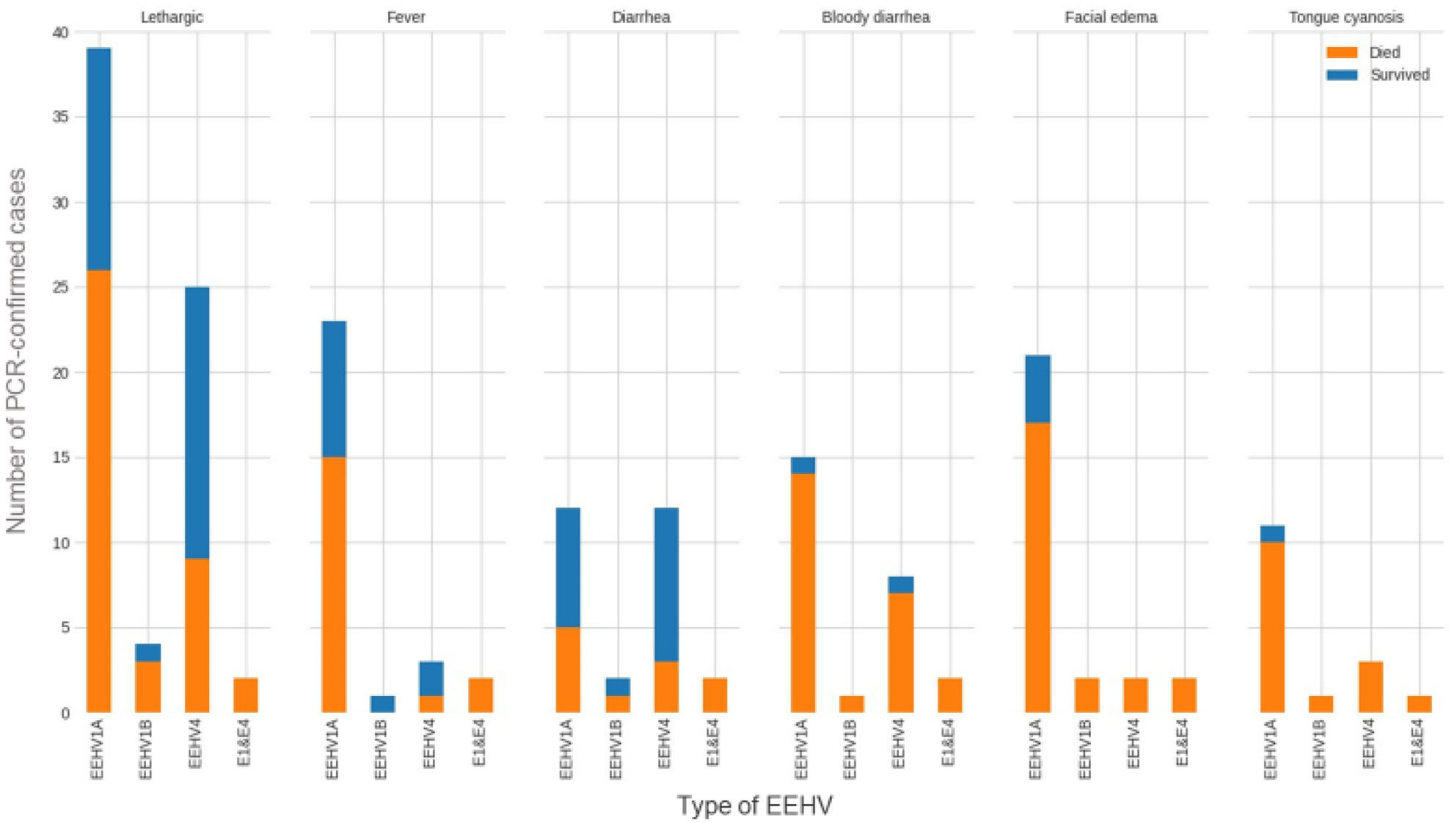
Clinical signs (lethargic, fever, diarrhea, bloody diarrhea, facial edema, and tongue cyanosis) presented during each type of EEHV infection (EEHV1A, EEHV1B, EEHV4, and EEHV1&4). The total number of cases was 83.

The most frequent clinical sign in the clinical group was lethargy, which presented in 84% of the cases. Other clinical manifestations appeared to differing degrees, including fever (37%), diarrhea (34%), bloody diarrhea (31%), facial edema (33%), and tongue cyanosis (20%). The two cases of EEHV1&4 co-infection displayed all of the clinical signs. Of note, EEHV1A&1B cases exhibited more signs of EEHV-HD, including bloody diarrhea, facial edema, and tongue cyanosis than those with EEHV4 (Figure 7).

### 3.4. Hematological findings

Of 24 cases with hematological profiles, seven were in the severe clinical group and all presented with thrombocytopenia, depletion of monocytes, lymphocytes and heterophils, a monocyte:heterophil (M:H) ratio lower than 2.37, hypoproteinemia (both albumin and globulin), severe grade of heterophil toxicity, and low red blood cell (RBC) counts and pack cell volumes (PCV). Three cases from this group (43%) had heterophil left-shifting, and one case (14%) presented with activated monocytes. The non-severe group included eight cases (5/8, 63%) that presented with similar clinical pathology to the severe group but of less severity, except no hypoproteinemia was found. Other clinical pathologies present in the non-severe group included activated monocytes (50%), heteropenia (25%), monocytosis (25%), reactive lymphocytes (25%), hypoproteinemia (25%), depletion of RBC count and PCV (25%), lymphocytosis (25%); only one case (12.5%) in this group had a normal blood profile. The subclinical group was comprised of nine cases, which presented with lymphocytosis. Reactive lymphocytes were found in seven cases (77.8%), while 4/9 (44.4%) responded with monocytosis and heterophil left-shifting, 33.3% with heterophilia, activated monocytes, and thrombocytopenia, and lastly 22.2% with heterophil toxicity (Table 1).

### 3.5. Treatment

From 81 cases with treatment data (NA = 22), 47 were received acyclovir or famciclovir and 34 cases went without treatment. In the non-severe clinical group, higher survival rates were observed with antiviral drug treatment. By contrast, survival was not affected by antiviral drug treatment in the severe clinical group. A higher survival rate was observed in calves treated with famciclovir (89%) compared to acyclovir (53%) treatment group (Table 2).

**Table 2.** Information on antiviral drug treatment in elephants with differing degrees of disease severity from 81 PCR-confirmed EEHV cases in Thailand.

Disease severity	No antiviral drug treatment		Acyclovir		Famciclovir	
	Died	Survived	Died	Survived	Died	Survived
Subclinical	0	9	0	2	0	1
Non-severe	3	2	0	16	0	6
Severe	17	3	18	2	1	1
Total	20/34 (58.8%)	14/34 (41.2%)	18/38 (47.4%)	20/38 (52.6%)	1/9(11.1%)	8/9 (88.9%)

## 4. Discussion

### 4.1. Demographic and clinical characteristics

This retrospective study is the first to describe demographic and clinical characteristics of EEHV in Thailand grouped by disease severity in 103 PCR-confirmed cases over a 14-year period from 2006 to 2019. The greatest number of cases was found in the north where the majority of captive elephants reside, with ∼37% being based at tourist camps in Chiang Mai province. We identified an increase in the number of cases and rates of survival over the last 4 years (since 2016), and while there were no surviving EEHV cases between 2006 and 2012. Between 2013 and 2019, there were 25 PCR-confirmed cases with clinical signs that responded to antiviral and other supportive treatments. Factors that have changed over the years include an increase in awareness of EEHV signs by elephant owners and mahouts (keepers), and more frequent PCR laboratory testing in young calves at the first indication of disease. An increase in survival within the clinical group after treatment (around 50% of cases) highlights the importance of early detection and treatment, knowledge of EEHV disease processes, and availability of highly dedicated veterinary practitioners throughout the country. In addition, antiviral and supportive drugs have become more available, which have played an important role in treating these cases (Sripiboon et al. 2017, 2020; Boonprasert et al. 2019). In 2019, there were 22 confirmed EEHV cases in Thailand out of 405 juvenile elephants aged 0–8 years, with a 50% survival rate, the highest to date.

About two-thirds of the cases were in the severe clinical group, one-third were non-severe, and less than 13% were subclinical. However, numbers might be underestimated, especially in the subclinical group, because it generally is only elephants with clinical signs that are tested. Most of the subclinical EEHV cases were the reinfected cases or had been identified by chance during routine health monitoring or were included in testing other infected EEHV elephants at the same facility. It is possible that some of the subclinical cases might have been due to reactivation of latent virus rather than primary infection. Using a luciferase immunoprecipitation assay to detect EEHV-specific antibodies, Fuery et al. (2020) found elephants with EEHV-HD were seronegative, while adult or otherwise healthy elephants were seropositive. Thus, it is possible that cases in the non-severe and subclinical groups in our study were reactivation cases, while cases in the severe group were primary infections. It is also known that adult elephants can be EEHV shedders through secretions without any clinical signs present (Stanton et al. 2010, 2013; Hardman et al. 2012; Atkins et al. 2013; Bennett et al. 2015; Azab et al. 2018; Bauer et al. 2018; Mahato et al. 2019).

Gender was not a significant demographic factor related to EEHV infection, only age. Over three quarters of cases were in the 0- to 4-year age group, with 49% observed in calves aged 2–4 years. We observed lower numbers of clinical EEHV cases, less severity of clinical signs and lower mortality in the >8- to 12-year age group, while there were no reports of severe clinical signs or mortality in elephants older than 12 years of age. In this study, the oldest case was an 18-year-old elephant with EEHV4 that exhibited non-severe clinical signs. Our results are similar to previous studies that showed the high-risk age group for EEHV is between 0 and 8 years of age, with a majority of cases emerging at 2–4 years (Bouchard et al. 2014; Long et al. 2016; Barman et al. 2017; Bennett 2018; Angkawanish et al. 2019; Boonprasert et al. 2019; Pavulraj et al. 2019). In general, calves in Thailand are weaned and go through a training process between 2 and 3 years of age, which corresponded to the highest numbers of EEHV cases. It is possible that these activities induce stress that may suppress the immune system, which then increases the susceptibility of these calves to EEHV infection (Barman et al. 2017; Bennett 2018). It might also be exacerbated by the loss of immunity afforded by transplacental maternal antibodies, which can protect against lethal EEHV-HD in elephant calves and has been shown to decline in calves after 2 years of age (Fuery et al. 2020).

Similar to previous report in Thailand (Sripiboon et al. 2016, 2020; Boonprasert et al. 2019) and from other parts of the world (Fickel et al. 2001; Schaftenaar et al. 2010; Atkins et al. 2013; Zachariah et al. 2013; Bouchard et al. 2014; Richman et al. 2014; Stanton et al. 2014; Seilern-Moy et al. 2016; Bennett 2018; Mahato et al. 2019; Oo et al. 2020), EEHV1A accounted for the majority of cases, with fewer attributed to EEHV4 and EEHV1B. EEHV1&4 co-infection does not appear to be common, with only two prior reports (Boonprasert et al. 2019; Seilern-Moy et al. 2016), and there were only two cases in our study group. Clinical signs were severe in these calves and both died. Although EEHV4 accounted for about a third of cases, more than 65% were subclinical or had non-severe clinical signs. There are limited reports on subclinical EEHV4 infections, while other reports find mainly EEHV1 in both subclinical and clinical groups (Stanton et al. 2010, 2014; Ackermann et al. 2017; Oo et al. 2020; Sripiboon et al. 2020).

### 4.2. Season

The highest number of EEHV cases were found in June-July, which are the months where the climate changes from hot and dry (summer) to warm and rainy (rainy season). There are no studies on how temperature or humidity affects health in elephants, but a study in dairy cows revealed low temperatures with high humidity can cause stress that affects milk production and may lead to infection of the respiratory tract or udder. By contrast, high temperature and low humidity may dehydrate mucous membranes, thus increasing the possibility of viral and bacterial infection (Herbut and Angrecka 2012).

### 4.3. Clinical outcomes

Lethargy and appetite loss were the most frequent signs observed and often are the ones most often noted by elephant owners or mahouts. Fever was reported in just over a third of the cases (37%) but might be underestimated because temperatures are difficult to obtain and usually only done during veterinary assessments or hospitalizations. In previous studies, apart from lethargy and appetite loss, sudden death is the most common clinical sign reported in EEHV cases (Zachariah et al. 2013; Long et al. 2016; Seilern-Moy et al. 2016). No studies have compared clinical outcomes between EEHV1A and EEHV1B. But in this study, both subtypes were associated with EEHV-HD resembling those in previous reports of EEHV1; i.e. facial edema, tongue cyanosis, bloody diarrhea, and sudden death (Muraro et al. 2017; Oo et al. 2020). Endothelial cells are involved in EEHV1 tissue tropism while intestinal organs are the target sites for EEHV4 (Seilern-Moy 2017; Kochagul et al. 2018) and a recent report indicated the major target cells of EEHV genomes in an acute infection were monocytes in blood vessels and macrophages of internal organs. These were preferentially distributed in the endothelia and smooth muscle cell of small blood vessels and microvessels mostly involved the cardiovascular system, which causes hemorrhagic disease in EEHV (Guntawang et al. 2020). However, severity of diarrhea was not different among the types of EEHV.

### 4.4. Hematological findings

The degree of clinical pathology was correlated with clinical outcomes. Monocytes/macrophages are carrier cells, while epithelial cells are infected cells for replication of EEHV genomes, causing apoptosis and extravasation of the peripheral blood monocytes (Srivorakul et al. 2019; Guntawang et al. 2020), a finding noted in this study. Leucopenia (monocyte, lymphocyte, and heterophil), M:H ratio < 2.37, thrombocytopenia, hypoproteinemia, a severe grade of heterophil toxicity, and anemia were observed in the severe clinical group and presented in around 25–63% of the non-severe clinical cases, similar to previous reports (Atkins et al. 2013; Pich et al. 2016; Fuery, Browning, et al. 2016; Fuery, Tan, et al. 2016; Stacy et al. 2017). Lymphocytosis and reactive lymphocytes were the first pathology signs, occurring in over three-quarters of animals in the subclinical group. Other pathologies such as monocytosis, heterophil left-shifting, heterophilia, activated monocytes, thrombocytopenia, and heterophil toxicity were also observed in this group, which means that even though there were no clinical signs present, changes in blood profiles were occurring, possibly from normal defense mechanisms in response to viral infection (Garner et al. 2009; Stacy et al. 2017; Wissink-Argilaga et al. 2019; Fuery et al. 2020).

### 4.5. Treatment

From the 47 EEHV cases that received antiviral drug treatment (acyclovir or famciclovir), 6.4% were subclinical, 47% were non-severe, and 47% were in the severe clinical group. In the severe clinical group, there was no difference in mortality between non-treated and treated elephants, and less than 12% survived. There was no mortality in treated elephants in the non-severe group. Possible reasons for unsuccessful treatment of EEHV-HD cases might be due to late diagnosis and irreversible cellular damage from the rapid progress of EEHV-HD by the time treatment was initiated. Although there was a higher percentage of survival cases with famciclovir treatment, data are too limited to conclude efficiency of any type of antiviral drug. In humans, famciclovir and acyclovir are effective in treating related diseases, like herpes simplex virus (HSV) and human cytomegalovirus (HCMV). In elephants, EEHV encodes both thymidine kinase (the usual target for famciclovir and acyclovir) and a serine/threonine protein kinase (the usual target for ganciclovir) similar to HSV and HCMV, but they have only 25% of both enzyme amino acids that identity with the orthologue target viruses for these drugs (Dastjerdi et al. 2016; Long et al. 2016), which might explain lower survival outcomes. In elephants, reports using famciclovir for EEHV clinical treatment found the active metabolite, penciclovir, in serum reached hypothetical levels for therapeutic effect in clinical ill cases based on human studies (Schmitt et al. 2000; Brock et al. 2012; Fuery, Browning, et al. 2016; Fuery, Tan, et al. 2016). However, the half-life of famciclovir in elephants is short at approximately 3 h and likely may not adequately control viral replication in EEHV cases (Dastjerdi et al. 2016). To date, only two pharmacokinetic studies have been conducted in Asian elephant calves, one with famciclovir (Brock et al. 2012) and the other with acyclovir (Khammesri et al. in preparation), so more work is needed. From previous reports, successful recovery outcomes of EEHV cases have been associated with antiviral drugs in combination with supportive treatment (Schmitt et al. 2000; Brock et al. 2012; Dastjerdi et al. 2016; Sripiboon et al. 2017; Bauer et al. 2018; Molenaar and Schaftenaar 2020), specifically famciclovir at doses of 8–15 mg/kg BW (PO/PR) tid-qid (Brock et al. 2012) and acyclovir at doses of 12 mg/kg BW (IV) bid (Sripiboon et al. 2017) and 45 mg/kg BW (PO) tid (Khammesri et al. in preparation). We recommend early diagnosis by PCR testing and treatment with antiviral drugs plus supportive therapies like intravenous fluid therapy, plasma transfusion, antibiotics, or vitamin C administration in clinical EEHV cases. Future studies of antiviral drug potency and efficacy in elephants are essential to help us improve the effectiveness of EEHV treatment.

## 5. Conclusions

This study was the first to compare clinical signs and outcomes across clinical severity groups. The findings support other studies that EEHV type 1 is the main cause of EEHV-HD with significant signs that include bloody diarrhea, facial edema, and tongue cyanosis. More than 80% mortality was observed when signs of EEHV-HD presented, and there were significant pathological changes like depletion of platelet counts less than 100 G/L, a monocyte:heterophil ratio < 2.37, hypoproteinemia, and severe anemia. In the non-severe group, EEHV type 4 infection was most commonly found with clinical signs that included lethargy and diarrhea, but no mortality. In the subclinical group, although there were no clinical signs, calves presented with changes in hematology, including lymphocytosis, reactive lymphocytes, monocytosis, heterophil left-shifting, heterophilia, activated monocytes, thrombocytopenia, and heterophil toxicity. Based on data from the previous 14 years in Thailand, elephant calves aged 2–4 years had the highest number of EEHV-HD cases and mortality. We recommended routine EEHV screening tests and blood profile monitoring, especially in high-risk age group (0- to 8-year old) to increases the effective rate of treatment for this disease. However, unlike many western facilities that have coordinated monitoring programs to track viral loads, few elephant calves are trained for blood collection in Thailand, and there is resistance or an inability to cover the cost of routine PCR analyses for early diagnoses. Limitations of our study were the retrospective nature of the design and the relatively small number of elephants despite having the largest number of PCR-confirmed EEHV cases. More studies on risk factors associated with severity of EEHV clinical signs, especially stress-related management like weaning, training, and transport, and proper data collection of medical and husbandry practices are required to help in predicting disease susceptibility and to better understand its epidemiology and clinical characteristics.
